# Construction of Double-Shelled Hollow Ag_2_S@Polydopamine Nanocomposites for Fluorescence-Guided, Dual Stimuli-Responsive Drug Delivery and Photothermal Therapy

**DOI:** 10.3390/nano12122068

**Published:** 2022-06-15

**Authors:** Minjie Gao, Zehua Han, Xu Zhang, Xueyan Zou, Lichao Peng, Yanbao Zhao, Lei Sun

**Affiliations:** 1 Engineering Research Center for Nanomaterials, Henan University, Kaifeng 475004, China; gaominjie8@126.com (M.G.); hanzehua0825@163.com (Z.H.); zouxueyan@henu.edu.cn (X.Z.); plc@henu.edu.cn (L.P.); zhaoyb902@henu.edu.cn (Y.Z.); 2 School of Pharmacy, Henan University, Kaifeng 475004, China

**Keywords:** hollow Ag_2_S nanospheres, mesoporous polydopamine, fluorescence imaging, photothermal–chemotherapy, smart drug delivery

## Abstract

The design and preparation of multifunctional drug carriers for combined photothermal–chemotherapy of cancer have attracted extensive attention over the past few decades. However, the development of simple-structured stimuli-responsive theranostic agents as both photothermal agents and chemotherapeutic agents remains a big challenge. Herein, a novel double-shelled nanocarrier composed of hollow Ag_2_S (HAg_2_S) nanospheres and a mesoporous polydopamine (MPDA) exterior shell was fabricated through a facile process. Notably, HAg_2_S possesses both fluorescence and photothermal properties. MPDA acts as a drug carrier and photothermal agent. Meanwhile, the cavity structure between HAg_2_S and MPDA provides more space for drug loading. The nanocarrier presents a high drug loading rate of 23.4%. It exhibits an apparent pH-responsive DOX release property due to the acidic sensitivity of PDA. In addition, the release of DOX is promoted under NIR irradiation, which is attributed to the heating action generated by the photothermal effect of HAg_2_S and MPDA. The cytotoxicity test shows that the nanocarriers possess good biocompatibility. Compared with single photothermal therapy or chemotherapy, the combined treatment represents a synergistic effect with higher therapeutic efficacy. In addition, the nanocarriers exhibit excellent fluorescence imaging capability and can target HepG2 cells. These simple-structured smart nanocarriers have a great potential for fluorescence-mediated combination cancer therapy.

## 1. Introduction

Nowadays, cancer is a major threat to the health of human beings. Photothermal therapy (PTT), which employs near-infrared (NIR) photothermal materials to ablate tumors, has become an extremely prospective anticancer strategy and attracted wide attention owing to its advantages, including negligible side effects and low systemic damage [1]. In particular, clinical trials of the first photothermal nanoparticle—PEGylated silica-cored Au nanoshells—had a great impact on modern tumor therapy [2]. Ablating tumors via mild-temperature PTT at the range of 43–48 °C was considered to be a promising strategy to inhibit the thermal tolerance of cancer cells and avoid damage to normal cells [3]. Currently, NIR photothermal materials mainly include organic materials, noble-metal-based nanomaterials, carbon-based nanomaterials, and semiconductor nanomaterials [4]. Among them, Ag_2_S nanomaterial is a typical direct narrow-bandgap semiconductor with both fluorescent and photothermal characteristics and has been successfully applied for cell labeling and photothermal therapy due to its unique NIR fluorescence emission, large photothermal conversion efficiency, excellent photostability, and low toxicity [5,6,7]. Recently, hollow Ag_2_S (HAg_2_S) nanospheres with unique cavity structures, excellent photothermal properties, and stable fluorescence performance have been investigated for fluorescence-mediated photothermal therapy [8].

However, monotherapy is normally not sufficient to generate an adequate therapeutic response, and PTT is no exception [9]. The combination of PTT and other therapies can generate improved treatment outcomes [10,11]. In particular, the combination of PTT, chemotherapeutic drugs, and smart nanocarrier-based drug delivery systems (DDSs) has been widely investigated due to their synergistic effect [12,13]. Currently, numerous nanocarriers have been utilized for DDSs, including polymers, liposomes, proteins, metal–organic frameworks (MOFs), and inorganic nanoparticles [14,15]. Mesoporous silica is often employed as a drug carrier in DDSs. Yang et al., reported the fabrication of a diversified nanoplatform (Ag_2_S@MSN-TGF) by encapsulating mesoporous silica on Ag_2_S nanoparticles, followed by loading the inside of the mesoporous silica with a hypoxia-active prodrug and coating the surface with glucose oxidase for the synergistic therapy of photothermal therapy and chemotherapy [16]. Zhao et al., developed a delivery system that was composed of Ag_2_S quantum dots coated with dendritic mesoporous silica, and the silica acted as a nanocarrier for localizing doxorubicin inside pores [17]. In order to enhance the bioavailability of chemotherapeutic drugs and minimize their toxic side effects, the designs of stimuli-responsive controllable drug release systems have been considered as a typical and successful strategy for chemotherapy [18,19]. However, as for mesoporous silica nanocarriers, it is a tedious process to construct stimuli-responsive smart gating through functionalized modification on their surface [20]. Consequently, there is a great need to develop a responsive drug delivery system with a facile preparation strategy and simple structure for photothermal–chemotherapy treatment.

Polydopamine (PDA) is an important component of melanin widely distributed in the human body and has obvious advantages of less toxicity [21]. Studies have demonstrated that PDA did not hinder the viability or proliferation of various cells such as osteoblasts and neuron cells [22,23]. PDA also plays an important role in regulating nerve cell state and repairing peripheral nerve and central nerve injury [24,25,26]. In addition, PDA possesses melanin-like molecular structures and can absorb and transform NIR light into heat for killing tumor cells, giving it potential for photothermal application [27,28]. In addition, PDA can be easily prepared under simple and mild conditions, and the abundant aromatic rings of PDA enable their surfaces to be loaded with dyes or chemical drugs via π–π stacking and/or hydrophobic–hydrophobic interaction [29]. Recent studies have demonstrated that these outstanding properties make PDA an optimal candidate for the combination of PTT and chemotherapy [30,31,32]. Compared with nonporous PDA, mesoporous polydopamine (MPDA) nanoparticles have a high drug loading capacity due to their mesoporous structure [33,34]. Specifically, PDA is extremely sensitive to acidity and has been employed extensively to establish responsive drug delivery systems for combined cancer treatment [35,36]. In addition, NIR has been demonstrated as an external stimulus to trigger drug release from nanocarriers via the photothermal effect of photothermal agents. Hence, PDA coating Ag_2_S can improve the shortcomings of a single treatment modality and enable controlled drug release. To the best of our knowledge, although the preparation of PDA-Ag_2_S nanoparticles has been already reported [37], the combined photothermal–chemotherapy of HAg_2_S@MPDA has not been explored until now.

**Figure 1:**
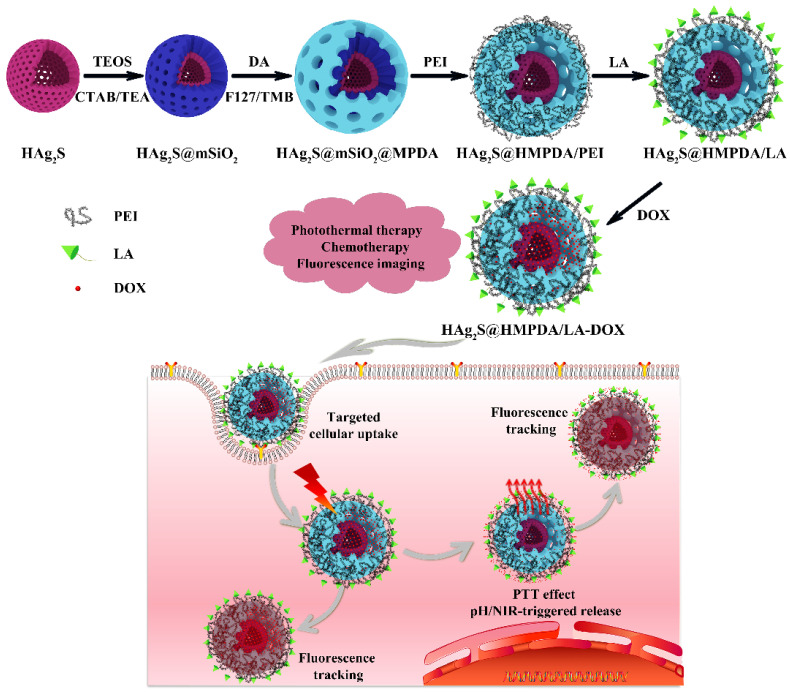
Schematic diagram of the fabrication of multifunctional double-shelled HAg_2_S@HMPDA/LA nanocarriers and their fluorescence-mediated combined photothermal chemotherapy for cancer treatment.

In this study, double-shelled HAg_2_S@MPDA nanocomposites were fabricated via a facile method with HAg_2_S nanospheres as the core and MPDA as the shell. The nanocomposites simultaneously perform three main properties of fluorescence imaging, photothermal therapy, and pH/NIR-responsive drug release. Figure 1 shows a schematic diagram of the fabrication of HAg_2_S@HMPDA nanocarriers and their fluorescence-imaging-mediated, combined photothermal chemotherapy for cancer treatment. Firstly, a mesoporous silica (mSiO_2_) shell was coated on the surface of HAg_2_S via the sol–gel reaction of tetraethyl orthosilicate (TEOS) with HAg_2_S nanospheres as the core and cetyltrimethylammonium bromide (CTAB) as the template agent. After removing CTAB molecules by extraction, HAg_2_S@mSiO_2_ nanoparticles were obtained. Afterward, pluronic F127 and 1,3,5-trimethylbenzene (TMB) were used as templating agents, and a layer of MPDA was generated on the surface of HAg_2_S@mSiO_2_ by the oxidative self-polymerization of dopamine (DA). The mesoporous HAg_2_S@mSiO_2_@MPDA nanoparticles were obtained by extracting and removing the template agent using ethanol and acetone as co-solvents. Then, PEI molecules were assembled on the surface of HAg_2_S@mSiO_2_@MPDA nanoparticles by electrostatic gravitational force. Simultaneously, the etching of SiO_2_ was achieved by the weak alkaline conditions provided by ethylene imine polymer (PEI), and the hollow double-shelled HAg_2_S@HMPDA/PEI nanoparticles were formed. The internal cavity structure can provide a large drug-loading space. Finally, lactic acid (LA), which can specifically bind to the receptor protein overexpressed on the surface of hepatocellular carcinoma cells, was grafted onto the MPDA shell, and the HAg_2_S@HMPDA/LA nanocarriers with ligand-receptor-mediated active targeting were obtained. The drug loading capacity and drug release behavior in vitro of nanocarriers were examined with doxorubicin hydrochloride (DOX) as a model drug. In addition, HAg_2_S nanoparticles endowed the nanocarrier with dual functions of fluorescence monitoring and photothermal therapy. The photothermal–chemotherapy synergistic effect for HAg_2_S@HMPDA/LA-DOX was investigated in vitro by thiazole blue (MTT) assay using HepG2 cells as the subject. The cellular uptake and targeting ability of the nanocarriers were examined through the fluorescence of HAg_2_S rather than exogenous fluorescent probes. This autofluorescence characteristic can be also utilized for biological imaging and monitoring the treatment process. Overall, this simple-structured and stimuli-responsive drug delivery system with fluorescent and photothermal properties has great potential in the precise treatment of cancer.

## 2. Materials and Methods

### 2.1. Materials

Polyvinylpyrrolidone (PVP K30) and silver nitrate (AgNO_3_) were purchased from Sinopharm Chemical Reagent Co., Ltd. (Shanghai, China). Sodium hydrate (NaOH), sodium carbonate (Na_2_CO_3_), sodium chloride (NaCl), triethanolamine (TEA), ammonia solution (NH_3_·H_2_O), CTAB, and methanol were provided by Tianjin Kermel Chemical Reagent Co., Ltd. (Tianjin, China). Absolute ethanol (EtOH) was obtained from Anhui Ante Food Co., Ltd. (Suzhou, China). LA, TEOS, DA, TMB, F127, PEI, 1-tetradecanol (TD), *N*-hydroxysuccinimide (NHS), sodium sulfate (Na_2_S·9H_2_O), *N*-(3-dimethylaminopropyl)-*N*′-ethylcarbodiimide hydrochloride (EDC), and MTT were obtained from Shanghai Aladdin Bio-Chem Technology Co., Ltd. (Shanghai, China). DOX was purchased from Shanghai Macklin Biochemical Co., Ltd. Fetal bovine serum (FBS) was provided by Hangzhou Sijiqing Bioengineering Material Co., Ltd. (Hangzhou, China). Dulbecco’s modified eagle medium (DMEM), Rhodamine 123 (Rh 123), trypsin-EDTA, phosphate-buffered saline (PBS), and dimethyl sulfoxide (DMSO) were purchased from Beijing Solarbio Science & Technology Co., Ltd. (Beijing, China). The HepG2 cell line was provided by Procell Life Science Technology Co., Ltd. (Wuhan, China). All reagents were directly used without any further purification.

### 2.2. Characterization

The morphology, microstructure, and element mapping of the samples were characterized using a JEM-F200 (JEOL, Tokyo, Japan) high-resolution transmission electron microscope (TEM), and element mapping was performed on a JED-2300 (JEOL, Tokyo, Japan) energy-dispersive X-ray spectroscope (EDX). The absorption and fluorescence spectra were obtained on a Cary 60 (Agilent, Santa Clara, CA, USA) UV-Vis spectrophotometer and a Cary Eclipse (Agilent, Santa Clara, CA, USA) fluorescence spectrophotometer, respectively. X-ray powder diffraction (XRD) spectra were obtained by a D8 Advance (Bruker, Karlsruhe, Germany) X-ray powder diffractometer. Size distribution and zeta potential distribution measurements were analyzed using a Zetasizer Nano ZS (Malvern, Malvern, UK) dynamic laser light scattering instrument (DLS). N_2_ adsorption/desorption isotherms were obtained on a Besorp-Max II (MicrotracBEL, Osaka, Japan) specific surface area and pore size analyzer. The surface area and the pore size distribution of the samples were calculated by the Brunauer–Emmett–Teller (BET) and non-local density functional theory (NLDFT) methods, respectively. The Fourier transform infrared (FTIR) spectra were recorded on a Vertex 70 (Bruker, Karlsruhe, Germany) Fourier transform infrared spectrometer with the wavenumber range of 4000–400 cm^−1^. The MDL-III-808 (New Industries, Changchun, China) laser equipment was used as a light source to measure the photothermal properties of samples. Thermal imaging was performed using an HM-TPH21Pro-3AQF (Hikvision, Hangzhou, China) handheld infrared thermometer (IRT). The cell activities were investigated using an 800 TS (BioTek Instruments, Vermont, US) enzyme-labeling instrument at the wavelength of 490 nm after an oscillation time of 10 min. Cell imaging was performed with an LSM 710 (ZEISS, Oberkochen, Germany) confocal laser scanning microscope (CLSM). Fluorescence quantitative analysis of cell uptake was performed on a FACSVerse (BD, Franklin Lakes, NJ, USA) flow cytometer (FCM).

### 2.3. Preparation of Mesoporous Silica-Coated Hollow Ag_2_S NPs (HAg_2_S@mSiO_2_)

Hollow Ag_2_S NPs were prepared using a template method according to our previous report [8]. The core–shell structure HAg_2_S@mSiO_2_ was prepared by a one-step method. Briefly, 1 mL of HAg_2_S nanoparticles, 55 mg of CTAB, and 100 μL of TEA were dispersed into a mixture solution containing 30 mL of distilled water and 4 mL of ethanol. After stirring for 1 h, 60 μL of TEOS (dispersed in 0.1 mL ethanol) was added dropwise and stirred for 6 h at 60 °C. Then, the raw products were purified by centrifugation and washing. Afterward, the products were refluxed with 60 mL of 8 mg/mL NaCl/methanol for 5 h to remove the template agent of CTAB by ion exchange. This process was performed at least 2 times. Finally, the HAg_2_S@mSiO_2_ nanoparticles were dispersed into 2 mL of absolute ethanol and stored at 4 °C for further use.

### 2.4. Preparation of MPDA-Coated HAg_2_S@mSiO_2_ Nanoparticles (HAg_2_S@mSiO_2_@MPDA)

Firstly, 500 mg of F127 and 50 mg of DA were dissolved into a mixture solution containing 5 mL of distilled water and 4 mL of ethanol. After the addition of 500 μL of TMB and dispersion by ultrasound, the solution color turned milky white. Secondly, it was mixed with 1 mL of HAg_2_S@mSiO_2_ suspension. After stirring for 1 h, 150 μL of NH_3_·H_2_O was added to trigger the polymerization of DA, and the reaction was allowed to proceed for 1 h at 25 °C. The product was separated by centrifugation at 9000 r/min for 5 min. Subsequently, 30 mL of a mixed solution of acetone/ethanol (volume ratio of 1:2) was used to remove F127 and TMB under sonication for 30 min, and this was repeated three times. Finally, HAg_2_S@mSiO_2_@MPDA composite nanospheres were obtained.

### 2.5. Preparation of HAg_2_S@HMPDA/PEI Nanoparticles

The as-prepared HAg_2_S@mSiO_2_@MPDA was dispersed in 20 mL of 15 mg/mL PEI solution and stirred for 3 h at room temperature. PEI was coated on the surface of PDA under the action of electrostatic attraction. At the same time, the mSiO_2_ layer was etched due to the weak alkalinity of PEI. The obtained product was centrifuged (9000 r/min, 5 min) and washed three times with distilled water to obtain double-shelled HAg_2_S@HMPDA/PEI nanoparticles.

### 2.6. Preparation of HAg_2_S@HMPDA/LA Nanoparticles

The target molecule LA was grafted on the surface of HAg_2_S@HMPDA/PEI nanoparticles via the coupling reaction of the carboxyl group (–COOH) and the amino group (–NH_2_) with the action of the EDC/NHS coupling agent. Firstly, 134 mg of LA was dissolved into 15 mL of PBS solution (pH = 7.4). Subsequently, 144 mg of EDC and 216 mg of NHS were added to the above solution. After stirring for 1 h at room temperature, 15 mL of HAg_2_S@HMPDA/PEI solution in PBS (pH = 7.4) was mixed with LA solution, and the reaction was continued at 30 °C for 8 h. The production was separated by centrifugation (9000 r/min, 5 min) and washed three times with distilled water. Ultimately, a targeted nanocarrier (HAg_2_S@HMPDA/LA) was obtained by freeze-drying.

### 2.7. Loading of DOX on HAg_2_S@HMPDA/LA (HAg_2_S@HMPDA/LA-DOX)

First, 20 mg of HAg_2_S@HMPDA/LA and 20 mg of DOX were added into 20 mL of water. After stirring for 48 h at room temperature, the mixture was centrifuged (9000 r/min, 5 min) and washed with water three times to remove the free DOX, and HAg_2_S@HMPDA/LA-DOX nanocomposites were obtained. The supernatant was gathered to estimate the drug loading capability (DLC). Two milliliters of the supernatant was sampled for UV–vis measurement at 480 nm wavelength to determine the concentration of the free DOX. The DLC was calculated according to Equation (1) as follows:

(1) $$\mathrm{DLC}\left(\%\right)=\frac{m_{0}-m_{free}}{m_{carrier}+m_{0}-m_{free}}\times 100\%$$

where *m*_0_ and *m_free_* are the masses of initial and free DOX, respectively, and *m_carrier_* is the mass of the HAg_2_S@HMPDA/LA nanocomposite.

### 2.8. Photothermal Performance of HAg_2_S@HMPDA/LA

To test the photothermal effects of the nanocarrier, 1.0 mL of HAg_2_S@HMPDA/LA (100, 150, 200, and 250 μg/mL) solution was irradiated with an 808 nm laser (0.5, 1.0, 1.5, and 2.0 W/cm^2^) for 10 min, and the solution temperature was recorded using a digital thermometer and an infrared thermal imager every 30 s.

To investigate the photothermal stability of the nanocarrier, 1.0 mL of 250 μg/mL HAg_2_S@HMPDA/LA solution was irradiated by an 808 nm laser at 1.5 W/cm^2^ for 10 min and naturally cooled to the room temperature for 5 cycles. The temperature variations were recorded.

### 2.9. In Vitro pH-Triggered Release of DOX from HAg_2_S@HMPDA/LA-DOX

Drug pH-triggered release tests were performed by the membrane dialysis method. Briefly, 5 mg of HAg_2_S@HMPDA/LA-DOX nanocomposites was suspended in 5 mL of PBS with various pH values (pH = 7.4, 6.5, 5.5). The suspension was injected into dialysis bags (MWCO 8000–14,000) and subsequently immersed in the corresponding releasing buffer medium (45 mL). The medium was constantly shaken (135 r/min) at 37 °C in the dark. At predetermined times, 1.0 mL of release solution was sampled and replaced with the same volume of fresh medium. The quantity of the released DOX was measured by using a UV-Vis spectrometer at the wavelength of 480 nm.

### 2.10. In Vitro NIR-Triggered Release of DOX from HAg_2_S@HMPDA/LA-DOX

NIR-triggered drug release behavior was estimated under various simulated temperatures under 808 nm laser irradiation. Since the NIR-triggered release is caused by the photothermal effect of the nanocarrier, the DOX release was carried out in PBS buffer (pH = 7.4) under various temperatures (25, 37, and 42 °C).

The DOX release with or without 808 nm laser irradiation test was carried out by dispersing 1 mg HAg_2_S@HMPDA/LA-DOX into 1.0 mL of PBS solution (pH = 7.4) under shaking (135 r/min) at 37 °C. At certain times, the suspensions were centrifuged, and 100 μL aliquots of the supernatants were sampled and equal volumes of fresh PBS solutions were added. For the laser irradiation group, the dispersion of HAg_2_S@HMPDA/LA-DOX was irradiated with an 808 nm laser (1.5 W/cm^2^) for 5 min every two hours. The released DOX in the supernatants at different time points was monitored with a UV-Vis spectrophotometer at 480 nm.

### 2.11. In Vitro Cytotoxicity Assay and Photothermal–Chemotherapy

MTT assay is used to detect cell proliferation, viability, and cytotoxicity by determining the mitochondrial ability to metabolize MTT. It has been generally performed to study antitumor efficacy in vitro [17,38]. The cytotoxicity of DOX, HAg_2_S@HMPDA/LA, and HAg_2_S@HMPDA/LA-DOX nanoparticles was measured using HepG2 cells. Typically, HepG2 cells were seeded in 96-well plates at a density of 5000 cells/well. After incubation at 37 °C and 5% CO_2_ atmosphere for 24 h, the culture medium was replaced with a fresh medium containing various concentrations of DOX, HAg_2_S@HMPDA/LA, and HAg_2_S@HMPDA/LA-DOX. In addition, pure culture medium and untreated cells were set as the blank control group and the negative control group, respectively. After incubation for another 24 h, 10 µL of 5 mg/mL MTT was added to each well, and the incubation was continued for 4 h. Afterward, the supernatant was abandoned, and 100 μL of DMSO was added to each well to dissolve the purple crystal. Subsequently, the viability was measured at 490 nm using a microplate reader.

To examine the synergistic effect of photothermal–chemo combined therapy, DOX, HAg_2_S@HMPDA/LA, and HAg_2_S@HMPDA/LA-DOX were cultured with HepG2 cells for 2 h. Subsequently, the cells were irradiated with an 808 nm laser (2.0 W/cm^2^) for 5 min. After further incubation for 24 h in the dark, the cell viability after therapy was tested by MTT assay.

### 2.12. In Vitro Targeting Ability of HAg_2_S@HMPDA/LA

To estimate the targeting ability of HAg_2_S@HMPDA/LA nanocarriers, cellular uptake assays were evaluated in HepG2 cells by FCM analysis with the fluorescence of HAg_2_S nanocores. HepG2 cells were plated into 6-well plates at a density of 1 × 10^5^ cells/well. After incubation for 24 h, the culture medium was replaced with a fresh medium containing 50 μg/mL HAg_2_S@HMPDA/LA and HAg_2_S@HMPDA/PEI. Untreated cells were set as the blank control group. After further incubation for 1, 2, and 4 h, HepG2 cells were digested with EDTA trypsin and then filtered into flow cytometry tubes. Ultimately, the fluorescence intensities of the nanocarriers ingested byHepG2 cells were quantitatively measured by FCM.

### 2.13. In Vitro Imaging of HAg_2_S@HMPDA/LA

HepG2 cells were seeded in laser confocal dishes at a density of 1 × 10^5^ cells. After 24 h incubation, the cells were further incubated with 50 μg/mL HAg_2_S@HMPDA/LA for 0.5, 1, and 2 h. Subsequently, the cells were cleaned with PBS and then stained with 1 mL of 5 μg/mL Rhodamine 123 for another 30 min. The cells were washed again three times and characterized under a confocal laser scanning microscope.

## 3. Results

### 3.1. Characterization of HAg_2_S@HMPDA/LA

**Figure 2:**
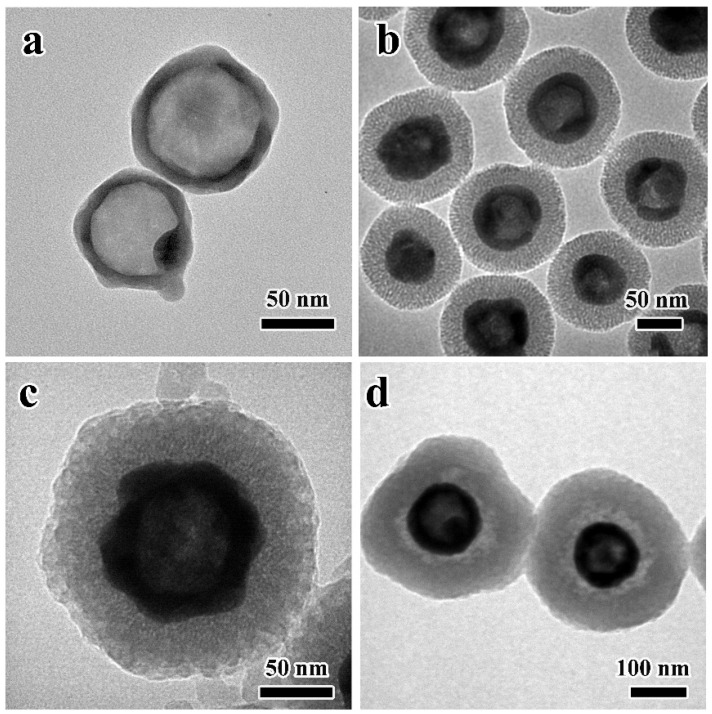
TEM images of HAg_2_S (**a**), HAg_2_S@mSiO_2_ (**b**), HAg_2_S@mSiO_2_@MPDA (**c**), and HAg_2_S@HMPDA/PEI (**d**).

Water-dispersible hollow Ag_2_S nanospheres with an average particle size of 70 nm were prepared, as shown in Figure 2a. After coating with mSiO_2_, a typical core–shell HAg_2_S@mSiO_2_ nanosphere with an average diameter of 125 nm can be clearly observed, as shown in Figure 2b, and the nanospheres have a shell thickness of 25 nm. Using F127 and TMB as template agents, a uniform layer of MPDA shell was deposited on the surface of HAg_2_S@mSiO_2_ nanosphere (as shown in Figure 2c) through the oxidative self-polymerization of dopamine and the interface co-assembly of TMB/F127 under alkaline conditions. The mesoporous structures of PDA were formed due to the π–π stacking interaction between the PDA structure and the π-electron-rich TMB molecules [39]. The HAg_2_S@mSiO_2_@MPDA nanosphere has an average particle size of 170 nm, and the outer MPDA shell thickness is about 20 nm. After the introduction of PEI, the mSiO_2_ shell was etched due to the weak alkalinity of PEI, and a clear double-shelled characteristic was exhibited through the striking contrast between shells and the cavity. Ultimately, the double-shelled HAg_2_S@HMPDA hollow nanospheres were obtained, as shown in Figure 2d. Meanwhile, the PEI molecule was assembled on the surface of HAg_2_S@HMPDA via electrostatic attraction to form the HAg_2_S@HMPDA/PEI nanocarrier. The mean particle size of the nanocarrier is about 210 nm. In addition, it can be clearly seen that there is an obvious gap between the two shells, and the mesoporous structure of the PDA shell becomes denser and thicker due to the coating of PEI. Furthermore, after the etching of mSiO_2_ with Na_2_CO_3_ but not PEI, the PDA layer obviously collapsed, and the mesoporous structure was also damaged, as shown in Figure S1. Thus, PEI was also proved to protect the overall structure of MPDA from damage and collapse.

**Figure 3:**
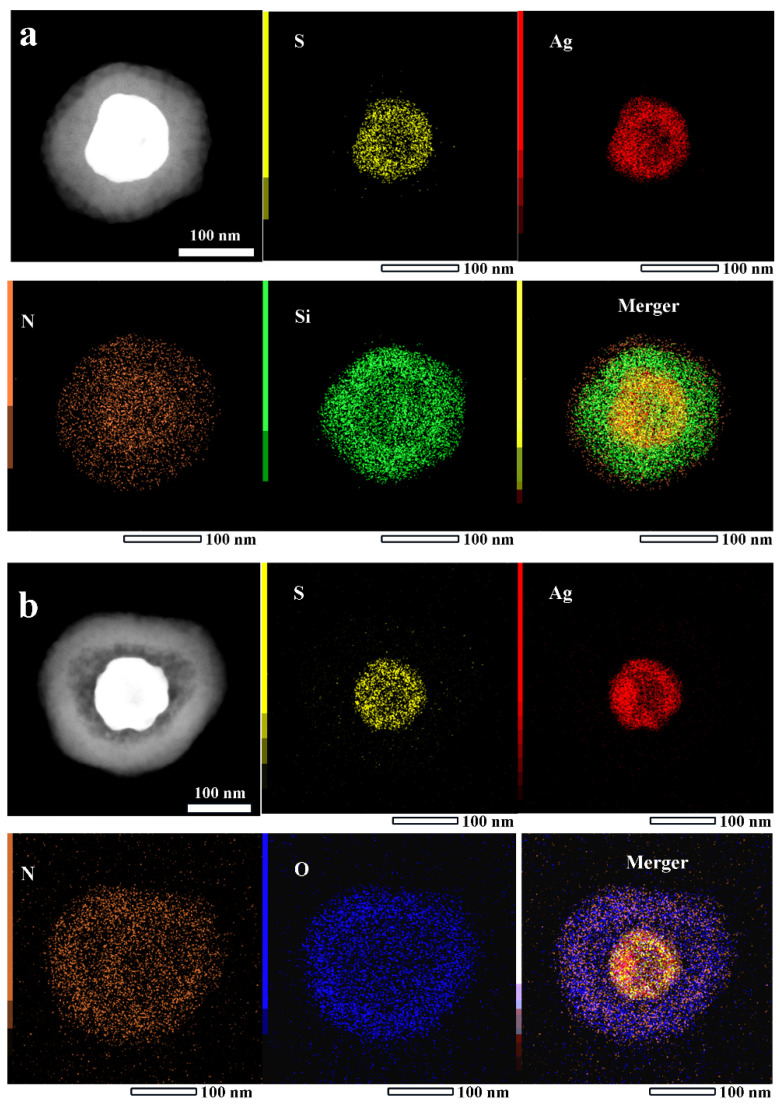
High-angle annular dark-field images and EDX elemental mappings of HAg_2_S@mSiO_2_@MPDA (**a**) and HAg_2_S@HMPDA/PEI (**b**); the scale bar is 100 nm.

To obtain the spatial distribution information of each element in the samples, EDX mapping observation was performed on HAg_2_S@mSiO_2_@MPDA and HAg_2_S@HMPDA/PEI nanoparticles, as shown in Figure 3. It can be seen from Figure 3a that Ag and S elements are uniformly distributed in the inner shell of the nanoparticle and there are obvious cavities in the center, indicating that the inner shell is composed of hollow Ag_2_S. The Si element is uniformly distributed in the outer layer of HAg_2_S and represents the SiO_2_ shell layer. It can also be seen that the distribution size of the N element is larger than that of the Si element, indicating that the PDA shell layer is distributed in the outer layer of SiO_2_. In addition, a clear contrast difference can also be observed from a high-angle annular dark-field image (HAADF), further confirming the three-layer core–shell structure. After the interaction with PEI, the mSiO_2_ layer is etched and the absence of the middle SiO_2_ layer can be clearly seen in Figure 3b. N and O elements are concentrated in the outer shell, indicating that the outermost shell layer is PDA and PEI. In addition, the contrast of the HAADF image also confirms the double-shelled structure. The EDX energy spectra (shown in Figure S2) indicate that N and O atomic ratios of HAg_2_S@mSiO_2_@MPDA and HAg_2_S@HMPDA/PEI are 15.57:49.56 and 71.46:18.31, respectively. Compared with the molecular formula C_8_H_16_NO_2_ of DA, the ratio of O element in HAg_2_S@HMPDA/PEI decreases sharply. This decrease is attributed to the etching of SiO_2_, and the ratio of the N element increases substantially due to the encapsulation of PEI. This result further confirms the encapsulation of the PEI layer.

The thickness of MPDA increased with the reaction time, as shown in Figure S3. DA firstly self-polymerized into small particles and was deposited on the surface of HAg_2_S@mSiO_2_ (as shown in Figure S3b). The mesopore structure was gradually formed under the synergistic effect of TMB and F127 (as shown in Figure S3c,d). With the prolongation of the reaction time, the DA molecules polymerized within the original mesoporous pore channels, causing the pore channels to gradually close (as shown in Figure S3e,f).

**Figure 4:**
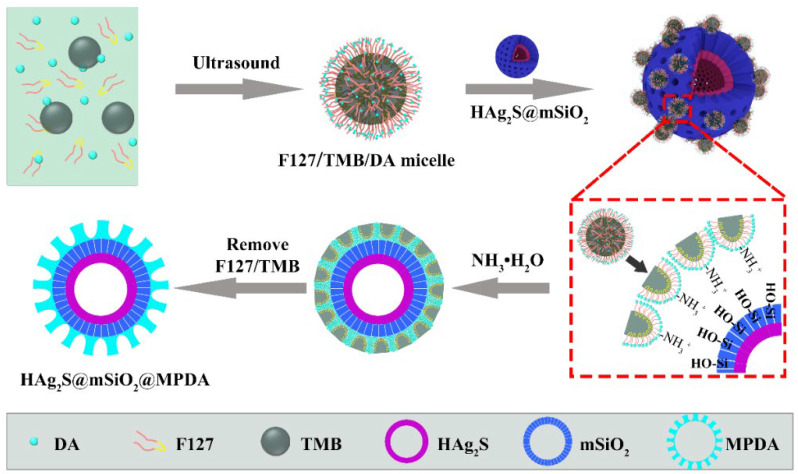
Illustration of the formation mechanism for HAg_2_S@mSiO_2_@MPDA nanoparticles.

The mechanism of the MPDA layer was also revealed, as shown in Figure 4. In the water–alcohol system, F127 and TMB were served as templates, and the MPDA layer was formed by the oxidation polymerization of DA. This reaction process occurred in the interface between the water and TMB. Since F127 is an amphiphilic copolymer, when F127, TMB, and DA were added into the system, an F127/TMB/DA micelle was formed [40,41]. The hydrophobic poly(propylene oxide) (PPO) unit of F127 was dispersed inside the hydrophobic TMB oil drop, while the hydrophilic poly(ethylene oxide) (PEO) unit was dispersed into the external aqueous phase of the TMB oil drop to form an oil-in-water droplet emulsion. The hydrophilic DA preferentially adsorbed on the surface of hydrophilic F127 and dispersed on the surface of oil droplets to form an F127/TMB/DA microemulsion system. F127 as a template guide was stably distributed on the oil–water interface of a microemulsion. When HAg_2_S@mSiO_2_ nanoparticles were added, droplets loaded with DA were rapidly adsorbed on the surface of the nanoparticles due to van der Waals force. After adding the catalyst of ammonia, DA molecules polymerized into small PDA particles under the action of oxygen, and these small particles gathered on the hydrophilic surface of F127 [42]. π–π stacking forces were formed between PDA and TMB due to their abundant benzene ring structures, resulting in the formation of mesoporous structure in PDA [39]. As the reaction proceeded, the DA-loaded droplets accumulated on the surface of HAg_2_S@mSiO_2_ nanoparticles. While the surface was completely covered, the droplets also moved close to each other under the induction of DA molecules to form a thicker PDA layer. Finally, MPDA-coated HAg_2_S@mSiO_2_ nanoparticles were obtained by removing TMB and F127.

The effect of catalyst addition was investigated on the formation of MPDA, as shown in Figure S4. When less ammonia was added (as shown in Figure S4a–c), the reaction was insufficient to generate abundant PDA particles for the formation of mesoporous structures. Hence, the PDA layer is irregular and the pore channels are not obvious. The thickness of the PDA layer increases significantly with the increase in ammonia addition (as shown in Figure S4e–f). However, the mesoporous pore channels of PDA are plugged due to the faster reaction. Therefore, the suitable catalyst content and appropriate reaction time are important factors for obtaining the ideal MPDA layer. As a result, the optimal parameter for the ammonia addition amount is 150 μL, and the optimal reaction time is 1 h.

**Figure 5:**
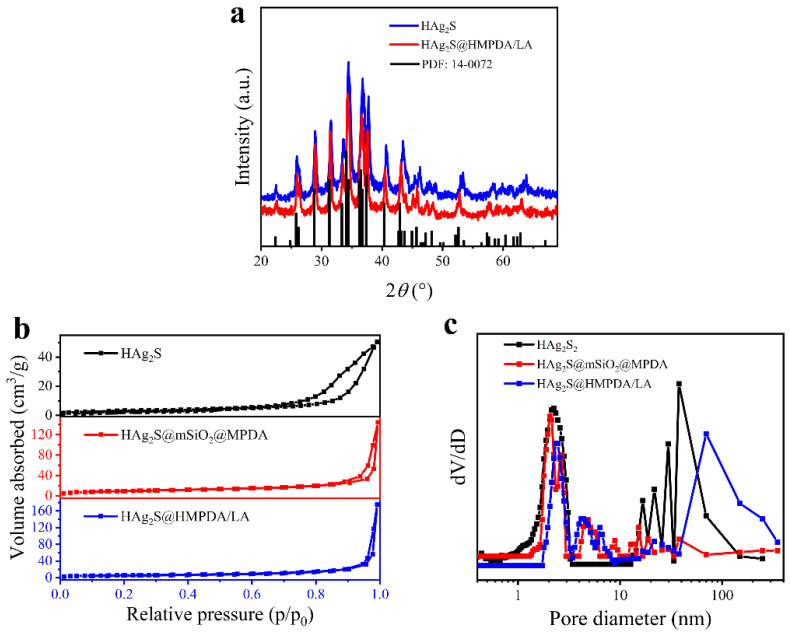
XRD patterns (**a**), N_2_ adsorption/desorption isothermals (**b**), and pore size distribution (**c**) of samples.

Figure 5a shows XRD patterns of the as-prepared HAg_2_S and HAg_2_S@HMPDA/LA samples. The diffraction peaks of samples are well matched with the standard line of monoclinic α-Ag_2_S (JCPDS No. 14-0072), indicating that the central core of the prepared drug carrier is composed of monoclinic phase α-Ag_2_S. The porous structure of the sample was investigated via BET gas adsorption measurements. Figure 5b,c show the N_2_ adsorption/desorption isotherms and the pore size distributions of HAg_2_S, HAg_2_S@mSiO_2_@MPDA, and HAg_2_S@HMPDA/LA nanoparticles. All the isotherms exhibit a type IV isotherm with a large hysteresis loop, which is characteristic of mesoporous materials. Such strong hysteresis at high relative pressures is related to the capillary condensation of large pore channels. In addition, the hysteresis loops with steep desorption branches are ascribed to the H2 (b) type, indicating the presence of an ink-bottle-like pore structure in the samples. This indicates that the samples contain large mesoporous structures, and these mesoporous structures are connected to the outer surface through smaller pores. The pore size distributions were calculated from the adsorption data using the nonlocal density functional theory (NLDFT) model, as shown in Figure 5c. For HAg_2_S nanospheres, pores of 38.3 and 2.2 nm are attributed to the hollow interiors and the porous shell, respectively, indicating that drugs can enter the inner cavity from the shell. As for HAg_2_S@mSiO_2_@MPDA nanoparticles, the appearances of the new pores with sizes of 2.0, 2.7, and 4.0~10.0 nm arise from the porous structures of mSiO_2_ and MPDA layers. As for HAg_2_S@HMPDA/LA nanocarriers, the large pores of around 73 nm are ascribed to the holes generated through the etching of the mSiO_2_ layer. Pores of various sizes and shapes are in accordance with those observed in the TEM image. The as-prepared HAg_2_S@HMPDA/LA nanocarriers exhibit a specific surface area of 24.49 m^2^/g and a pore volume of 0.25 cm^3^/g, respectively, higher than those of HAg_2_S nanospheres (10.98 m^2^/g and 0.077 cm^3^/g) and other reported MPDA nanoparticles [34,43]. These characteristics are conducive to drug loading.

**Figure 6:**
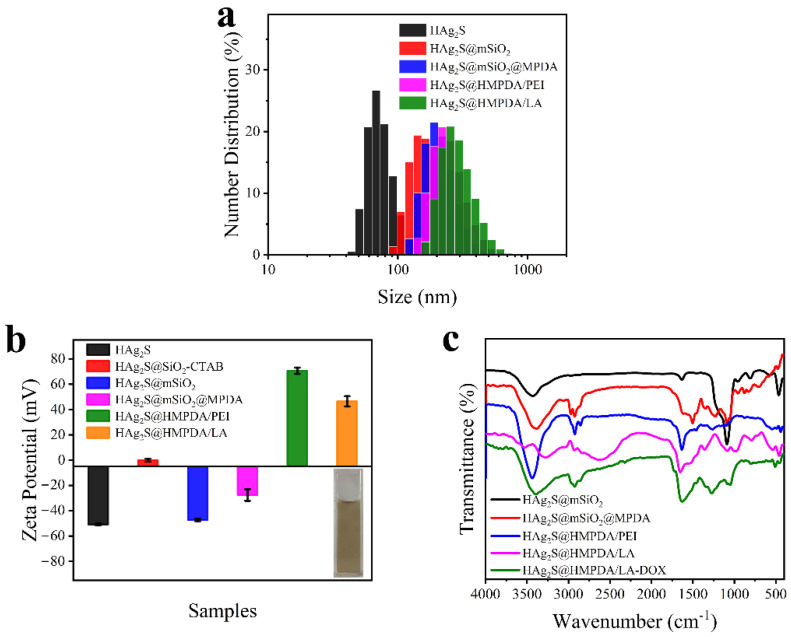
Particle size distributions measured by DLS (**a**); zeta potentials (**b**), with an inserted photo showing the colloidal stability of HAg_2_S@HMPDA/LA aqueous solutions; and FTIR spectra (**c**) of different samples.

DLS and zeta potential of the samples in each step of preparation were monitored to confirm the success of each reaction step. The average diameters of HAg_2_S, HAg_2_S@mSiO_2_, HAg_2_S@mSiO_2_@MPDA, HAg_2_S@HMPDA/PEI, and HAg_2_S@HMPDA/LA measured by DLS are 68, 151, 191, 223, and 256 nm, respectively (as shown in Figure 6a). This increasing trend was consistent with the TEM observation result. There is a distinction between the sizes measured by DLS and TEM. In general, DLS presents a hydrodynamic size related to the hydration layer in the hydrated state, whereas TEM shows the actual size of the sample in the dry state. Hence, the DLS test result usually shows a larger particle size than TEM.

The zeta potentials of the corresponding samples at each step were also measured in distilled water. As shown in Figure 6b, the zeta potential of HAg_2_S nanospheres is −50.7 mV. With the help of the cationic template CTAB, an mSiO_2_ layer was generated on the surface of the nanospheres with a negative zeta potential of −47.2 mV due to the external silicon hydroxyl group. After being coated with MPDA, HAg_2_S@mSiO_2_@MPDA nanoparticles are negatively charged (−27.5 mV) due to deprotonation of the phenolic group [44]. It is noteworthy that the zeta potential of HAg_2_S@mSiO_2_-CTAB nanoparticles is 0 mV when CTAB is not removed. In this case, DA cannot self-polymerize on the SiO_2_ surface. It is further confirmed that F127/TMB/DA micelles are aggregated on the SiO_2_ surface via van der Waals forces. When PEI is introduced, the potential of HAg_2_S@MPDA@PEI nanoparticles increases to a positive value of 70.7 mV owing to the plentiful amino groups in PEI. After grafting with the targeting molecule of LA, the zeta potential decreases to 46.5 mV, which is attributed to hydroxyl groups of LA. In general, nanoparticles with a zeta potential above 30 mV or below −30 mV are colloidally stable [45,46]. In addition, the photo inserted in Figure 6b shows that HAg_2_S@HMPDA/LA exhibits good dispersion in aqueous solutions. The positive charge of the sample can enhance the cellular uptake towards cancer cells through the electrostatic interaction with the negatively charged cell membrane. Therefore, the as-prepared nanocarriers are suitable for the field of cancer therapy.

The successful reaction of each prepared step was further confirmed by FTIR spectra. As shown in Figure 6c, the HAg_2_S@mSiO_2_ nanoparticles display three absorption peaks at 3432, 1081, and 804 cm^−1^, ascribed to O–H stretching vibration of silanol groups, Si−O−Si asymmetric stretching, and Si−O−Si symmetric stretching, respectively [47]. After the coating with PDA, the emerging absorption bands at 1450, 1500, 1574, and 1617 cm^−1^ are attributed to the stretching vibration of the benzene ring skeleton (C=C), which is an important feature for the identification of MPDA. The strong and broad absorption band at 3386 cm^−1^ is assigned to the stretching vibration of primary amines in MPDA and overlaps partially with the absorption peak of Si–OH. The peaks at 2923 and 2858 cm^−1^ are attributed to the stretching vibration of −CH− in MPDA. An absorption peak at 1227 cm^−1^ is generated by the stretching vibration of the phenolic hydroxyls. For HAg_2_S@HMPDA@PEI, the absence of the peaks at 1081 and 804 cm^−1^ demonstrates the etching of SiO_2_. The strong absorption peak at 3441 cm^−1^ is attributed to the stretching vibration of −NH− in PEI. The absorption peaks at 1634 and 2858 cm^−1^ are significantly enhanced due to the presence of substantial amino and alkyl groups in PEI. In the spectra of HAg_2_S@HMPDA/LA, the peaks at 3534 and 3278 cm^−1^ are due to the stretching vibrations of N−H in the primary amide and hydrogen-bonded O–H. The peaks at 980 and 1082 cm^−1^ are ascribed to the symmetric and asymmetric stretching vibrations of the cyclic ether bond in LA. The peak at 1651 cm^−1^ is assigned to the stretching vibrations of C=O in the amide bond. These peaks demonstrated the formation of amide bonds, indicating the successful grafting of LA. As for HAg_2_S@HMPDA/LA-DOX, the strong and wide absorption peak at 1640 cm^−1^ is caused by the stretching vibration of the benzene ring skeleton and C=O in DOX, demonstrating the successful loading of DOX.

Figure S5a shows the UV-Vis absorption spectrum of DOX, HAg_2_S, HAg_2_S@HMPDA/LA, and HAg_2_S@HMPDA/LA-DOX aqueous solutions. It is seen that the absorption bands of the as-prepared nanoparticles extend to the NIR region. This provides a theoretical basis for their application in the field of NIR photothermal therapy. After coating with the PDA layer, the absorption intensity of HAg_2_S@HMPDA/LA is significantly increased due to strong NIR absorption of PDA [48]. In the spectrum of HAg_2_S@HMPDA/LA-DOX, a new broad absorption peak appears at 490 nm compared with HAg_2_S@HMPDA/LA nanocarriers, indicating the successful loading of the DOX drug. DOX drug is not only loaded into the mesoporous channels but also can be present on the surface of nanocarriers via π−π-conjugated or hydrogen-bonded interaction between PDA and DOX [20]. The red-shifted (from 480 to 490 nm) absorption peak of DOX further confirms π–π stacking interaction between DOX and PDA [49]. The fluorescence spectrum (Figure S5b) shows that the HAg_2_S@HMPDA/LA nanocarrier emits a narrow and symmetric NIR fluorescence peak at 820 nm owing to the HAg_2_S nanocore. The emission peak of the nanocarrier is almost unchanged after the loading of drugs, indicating that the addition of DOX negligibly affects the fluorescence intensity of the nanocarriers. Therefore, the as-prepared nanocarrier has the potential to be used as a fluorescent probe for in vivo imaging.

### 3.2. Photothermal Property of HAg_2_S@HMPDA/LA

**Figure 7:**
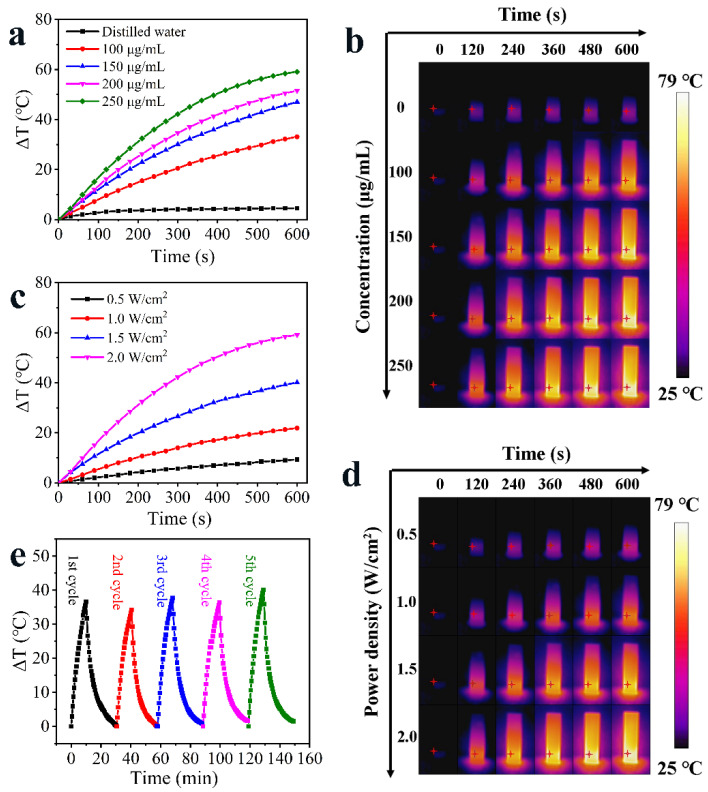
Temperature elevation curves of HAg_2_S@HMPDA/LA nanocarriers with various concentrations under 808 nm irradiation (2 W/cm^2^) (**a**) and the related IRT images (**b**); temperature elevation curves of HAg_2_S@HMPDA/LA nanocarriers (250 µg/mL) at various irradiation power densities of 808 nm irradiation (**c**) and the related IRT images (**d**); temperature variation curves of HAg_2_S@HMPDA/LA nanocarriers under five irradiation/cooling cycles (**e**).

The photothermal ability of HAg_2_S@HMPDA/LA nanocarrier was investigated by monitoring the temperature variation under 808 nm laser irradiation, as shown in Figure 7a. The results show that the temperature of HAg_2_S@HMPDA/LA solution (250 μg/mL) increases by 59.1 °C after 10 min irradiation with 2.0 W/cm^2^ NIR light and exhibits a concentration-dependent photothermal effect. In contrast, the temperature of the control group of pure water only increases by 4.5 °C, indicating that the as-prepared nanocarrier possesses good photothermal performance. The photothermal effect of HAg_2_S@HMPDA/LA was also found to be dependent on the power density of the laser source (Figure 7c). The temperature rise rate increases with the increase in power density. When the power density is 2.0 W/cm^2^, the temperature increases rapidly by about 10 °C within 1 min. Studies have shown that cancer cells can be killed by exposure to 41–47 °C for a few minutes due to their poor heat resistance, while normal cells are almost unaffected. As the normal temperature of the human body is 37 °C, the temperature will reach the effective temperature for killing cancer cells when increased by 9 °C. In addition, the temperature of HAg_2_S@HMPDA/LA nanocarrier can be regulated in control by adjusting the concentration of nanocarriers, irradiation power density, and irradiation time to minimize the damage to normal tissues. It is observed by infrared thermography that HAg_2_S@HMPDA/LA nanocarrier has a quite sensitive NIR response. As shown in Figure 7b,d, there is a significant change in temperature only at the irradiated liquid location, and the surrounding temperature change is mainly due to heat conduction, thus ensuring that the temperature at the unirradiated location is maintained within the normal range. Additionally, the significant thermal contrast indicates that the nanocarrier has infrared thermography (IRT) characteristics. Since repeated laser irradiation is often required in cancer treatment, an ideal photothermal material should also have excellent photothermal stability. Hence, the photothermal stability of the HAg_2_S@HMPDA/LA nanocarrier was investigated by repeated irradiation with an 808 nm laser. The temperature of the nanocarrier shows no significant decrease after successive laser irradiation for five cycles (Figure 7e). Consequently, the as-prepared nanocarriers have an excellent photothermal effect and photostability for photothermal therapy.

### 3.3. pH/NIR-Responsive Drug Release Activities

**Figure 8:**
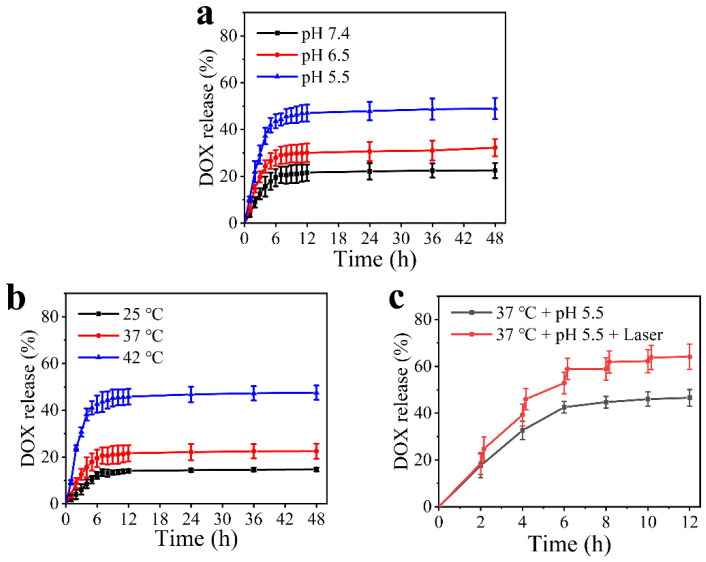
Release profiles of DOX from HAg_2_S@HMPDA/LA-DOX in corresponding release environment: different pH values (**a**), various temperatures (**b**), and with or without laser radiation (**c**).

The HAg_2_S@HMPDA/LA nanocarrier has a mesoporous and microporous structure, which is conducive to drug loading and sustained release. The drug DOX was chosen as the model to investigate the drug loading and release ability of the nanocarrier. The corresponding drug loading capacity was 23.4%. It was a comparatively high loading value in contrast to that of other reports [16,17]. Due to the pH-responsive properties of PDA, the drug release from HAg_2_S@HMPDA/LA-DOX was measured at different pH (pH 7.4, 6.5, and 5.5), as shown in Figure 8a. The drug release is accelerated with the decrease in pH value. When this delivery system is under normal physiological conditions (pH 7.4), the drug release reaches the equilibrium state at 6 h and exhibits an accumulative release amount of 22.5% for 48 h. When the pH value is increased to 6.5, the total drug release reaches 32.3%. Contrastingly, the total release amount is 49.0% at pH 5.5, almost 2.4 times higher than that at pH 7.4, showing a significant enhancement. The results indicate that the lower pH facilitates the release behavior of DOX. The acidity-promoted DOX release from HAg_2_S@HMPDA/LA-DOX is attributed to two factors. On the one hand, the protonation of amine groups in PDA and DOX molecules in acidic conditions partially weakens the π–π interactions between PDA and DOX, triggering the drug release [50]. On the other hand, it is widely believed that the structure of PDA mainly consists of oligomers via hydrogen bonding or π–π stacking. Under acidic conditions, PDA will form an unstable state and have a certain degree of degradation, which has been previously reported [20]. All the release profiles exhibit a classic biphasic release pattern: an initial abrupt release and a subsequent sustained release. The initial abrupt release of DOX is primarily attributed to the release of DOX adsorbed on the outer layer of the nanocarriers. The sustained release is due to the diffusion-mediated release from nanocarriers. This sustained release characteristic of the as-prepared nanocarriers agrees with other previously reported π–π stacking-based drug-controlled systems [49,50].

The drug release of HAg_2_S@HMPDA/LA-DOX nanoparticles can also be triggered by NIR due to the photothermal effect of HAg_2_S and PDA. To investigate the NIR-responsive drug release, the release profiles were first monitored under different temperatures, as shown in Figure 8b. It is seen that the drug release rate of HAg_2_S@HMPDA/LA-DOX nanoparticles becomes faster with the increase in temperature. When the temperature reaches 42 °C, a maximum drug release rate of 47.5% is obtained. Then, the NIR-stimulated responsive release of the drug was carried out with and without laser irradiation. As shown in Figure 8c, when HAg_2_S@HMPDA/LA-DOX nanoparticles are irradiated with 808 nm laser at pH 5.5, the drug release amount and rate are significantly increased. Compared to the control group (without laser irradiation), the DOX-release percentage with laser irradiation increased by 17.4% and eventually reached as high as 64.0%, indicating that the photothermal effect of HAg_2_S and PDA can further promote the DOX release. This may be ascribed to the stimulation of the heat generated by the photothermal effect, promoting the diffusion of DOX into the release medium. Hence, the as-prepared pH- and NIR-responsive nanocarriers can minimize early drug leakage during blood circulation and improve the drug release in an acidic tumor environment, which can effectively promote the drug delivery efficiency and reduce the toxic side effects on normal tissues.

**Figure 9:**
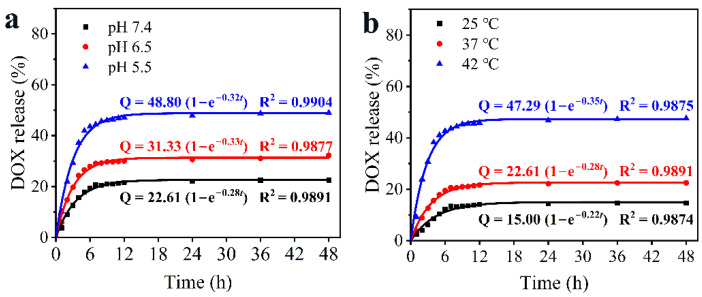
First-order kinetic models of DOX released from HAg_2_S@HMPDA/LA-DOX at different pH values (**a**) and temperatures (**b**).

To further illustrate the release behavior of the HAg_2_S@HMPDA/LA-DOX sample, zero-order, first-order, and Higuchi release kinetic models were used to fit the DOX sustained release profiles of the sample under different release conditions. As shown in Table S1, the release behaviors of DOX in various pH values and temperatures are well fitted with the first-order kinetic model according to the fitting results and the correlation coefficient (R^2^) values. The first-order kinetic equation is expressed as follows:

(2) $$Q_{t}{=Q}_{\max}{\;(1\;-\;e}^{-kt})$$

where Q*_t_* is the drug release at time *t*, Q_max_ is the maximum release, and k is the release rate constant of the first-order kinetic model. Figure 9 shows the curves plotted for the first-order kinetic model and the corresponding parameters fitted.

### 3.4. In Vitro Cytotoxicity Assay and Synergistic Photothermal–Chemotherapy

**Figure 10:**
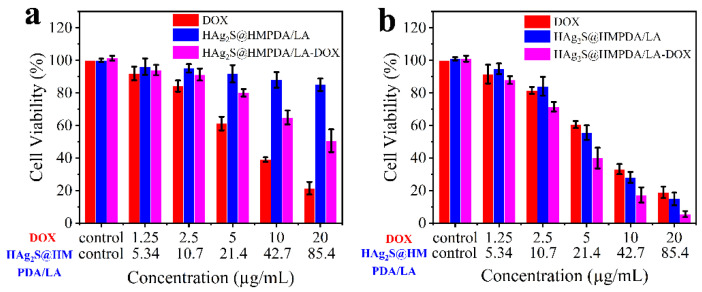
Relative viabilities of HepG2 cells incubated with DOX, HAg_2_S@HMPDA/LA, and HAg_2_S@HMPDA/LA-DOX without laser (**a**) and with laser (**b**).

The biocompatibility of drug carriers is an essential parameter to evaluate. The MTT assay was carried out to evaluate the biocompatibility of the samples with HepG2 as the cell model, and the results are shown in Figure 10a. It is seen that when the concentration of the HAg_2_S@HMPDA/LA nanocarrier is as high as 85.4 μg/mL, the cell viability remains above 85%, indicating that the carrier has good biocompatibility. As DOX is loaded into the carrier, the cell viability is reduced to 50.6%. This demonstrates that the drug-loaded system exhibits an apparent chemotherapeutic effect. However, compared to the free DOX (about 21.4% of cell viability), the cytotoxicity of the drug-loaded system is significantly lower, which could reduce the toxic side effects on normal tissues.

The combined treatment effects of samples in vitro were also evaluated by MTT assay. HepG2 cells containing DOX, HAg_2_S@HMPDA/LA, and HAg_2_S@HMPDA/LA-DOX were irradiated with an 808 nm laser for 5 min and further incubated for 24 h. The cell viabilities are shown in Figure 10b. It can be observed that the cell viabilities decreased significantly with the increase in the concentration of HAg_2_S@HMPDA/LA. When the concentration is 42.7 μg/mL, the cell viabilities decrease to 33.2%, indicating its apparent photothermal killing effect on HepG2 cells. In addition, the HAg_2_S@HMPDA/LA nanocarrier exhibits a slightly inferior cell viability to that of DOX after irradiation, showing that the nanocarrier can perform effective tumor therapy. This is attributed to the fact that both Ag_2_S and PDA can convert NIR light energy into heat energy, thereby ablating cancer cells through photothermal treatment. As for DOX, cell viability is almost unaffected with laser irradiation, compared with Figure 10a. For the HAg_2_S@HMPDA/LA-DOX photothermal–chemotherapy group, the cell viability of HepG2 is significantly lower than that of the single chemotherapy group (HAg_2_S@HMPDA/LA-DOX without laser) and photothermal treatment group (HAg_2_S@HMPDA/LA with laser). When the concentration of HAg_2_S@HMPDA/LA-DOX is 42.7 μg/mL (equivalent to 10 μg/mL DOX), the cell viability is only 17.3%. In contrast, the corresponding cell viabilities are 64.9% and 28.1% for the chemotherapy and photothermal treatments alone, respectively. These results suggested that the synergistic photothermal therapy and chemotherapy is superior to a single treatment. Therefore, the construction of HAg_2_S@HMPDA/LA nanocarriers is capable of achieving the synergistic treatment of photothermal therapy and chemotherapy.

### 3.5. In Vitro Cancer Cell Targeting Effect

**Figure 11:**
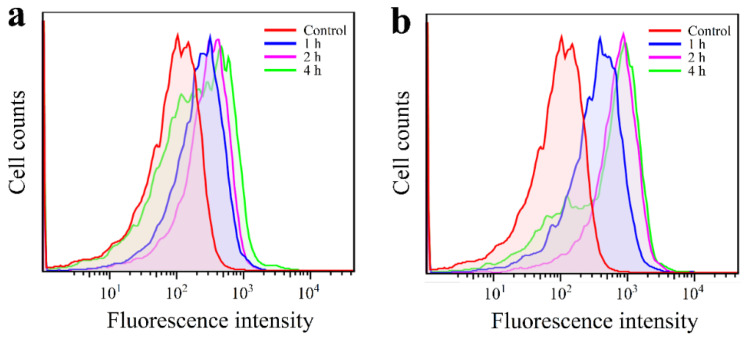
FCM analysis of HepG2 cells after incubation with HAg_2_S@HMPDA/PEI (**a**) and HAg_2_S@HMPDA/LA (**b**).

As the core of the HAg_2_S@HMPDA/LA nanocarrier, HAg_2_S can emit a fluorescence peak at 820 nm. Thus, this feature can be directly exploited to measure the targeting of the nanocarrier to cancer cells and its intracellular distribution. As a small targeting molecule, LA can be specifically recognized by the overexpressed asialoglycoprotein receptors on hepatocellular cancer cell membranes. Therefore, the carboxyl group of LA was activated by the coupling agent EDC/NHC and then reacted with the amino group of PEI on the surface of HAg_2_S@HMPDA/PEI nanocarriers to form an amide bond. LA-modified actively targeted double-shelled nanocarriers were thus obtained. The targeting effect of HAg_2_S@HMPDA/LA nanocarriers on HepG2 cells with high expression of asialoglycoprotein receptors was investigated by flow cytometry with HAg_2_S@HMPD/PEI (without grafting LA) as the control, as shown in Figure 11. Flow cytometry analysis shows that the fluorescence intensity in HepG2 cells gradually increases with the incubation time, which implies that the cellular uptake of HAg_2_S@HMPDA/LA and HAg_2_S@HMPDA/PEI gradually increases. In addition, the fluorescence intensity in HepG2 cells incubated with HAg_2_S@HMPDA/LA (Figure 11b) is significantly higher than that with HAg_2_S@HMPDA/PEI (Figure 11a) within the same incubation time, indicating that LA significantly enhances cellular uptake of HAg_2_S@HMPDA/LA compared to that of HAg_2_S@HMPDA/PEI. This is because LA can specifically bind to the asialoglycoprotein receptors that are highly expressed on the surface of HepG2 cells, allowing the nanocarriers to internalize into HepG2 cells through receptor-mediated endocytosis.

### 3.6. Cellular Uptake and Imaging

**Figure 12:**
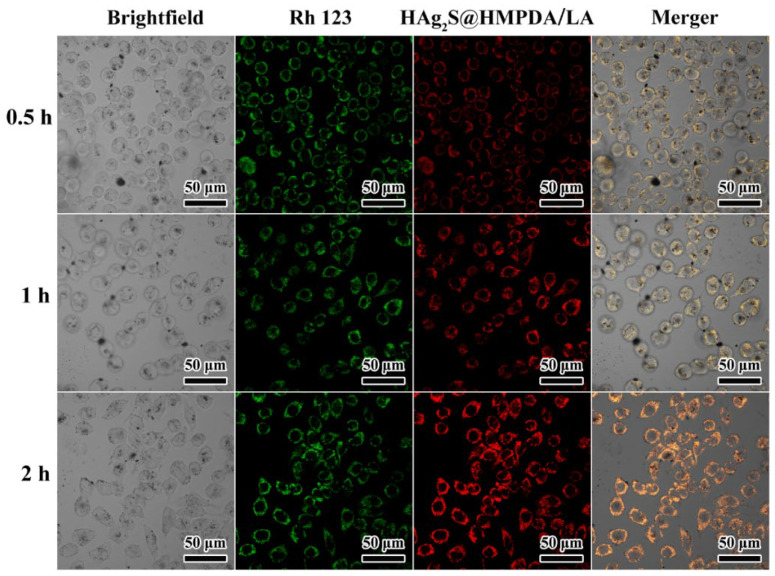
CLSM of HepG2 cells’ incubation with HAg_2_S@HMPDA/LA for 0.5, 1, and 2 h.

Effective intracellular uptake of nanocarriers is a prerequisite for the realization of their therapeutic functions. To investigate further the intracellular distribution of HAg_2_S@HMPDA/LA nanocarriers, the fluorescence signal of HAg_2_S in HepG2 cells was tracked through CLSM, as shown in Figure 12. The green fluorescence represents the cytoplasm stained with Rhodamine 123, while the red fluorescence originates from the HAg_2_S core of the prepared nanocarriers. Furthermore, the intensity of red fluorescence gradually increases with the incubation time from 0.5 h to 2 h, indicating the time-dependent cellular uptake of nanocarriers. This result is consistent with that of the above flow cytometry analysis. It also can be observed from Figure 12 that the fluorescence signal of nanocarriers was mainly detected in the cytoplasm region according to the locations of the green and red fluorescence. Therefore, the as-prepared HAg_2_S@HMPDA/LA nanocarriers can utilize their fluorescent properties for cell imaging and are expected to be used for diagnosis and monitoring of cancer.

## 4. Conclusions

In summary, a multifunctional double-shelled HAg_2_S@HMPDA/LA smart drug delivery nanocarrier was prepared with a simple and efficient strategy. HAg_2_S nanospheres, which served as the core, have both fluorescent imaging and photothermal properties. As the outer shell, the MPDA acted as both a drug carrier and a photothermal therapeutic agent. In addition, the cavity between Ag_2_S and MPDA provides space for drug loading. In addition, the modification of LA significantly enhanced the uptake of the nanocarrier by HepG2 cells. As a result, the obtained nanocarriers possess a high drug loading capacity (23.4%) for DOX and excellent photothermal performance. Their drug release exhibits pH- and NIR-responsive release behavior. Importantly, the carrier achieves synergistic photothermal and chemical treatment of cancer cells and can enter the cytoplasm for cell imaging. The above results illustrate the feasibility of utilizing HAg_2_S@HMPDA/LA-DOX nanocomposites as fluorescent-imaging-mediated photothermal chemotherapeutic agents for cancer treatment.

## Data Availability

The data presented in this study are available on request from the corresponding author.

## Supplementary Materials

The following supporting information can be downloaded at: https://www.mdpi.com/article/10.3390/nano12122068/s1, Figure S1: TEM images of HAg_2_S@HMPDA obtained after the etching with different concentrations of Na_2_CO_3_: (a–d) 0.05, 0.10, 0.20, 0.40 mmol/L; Figure S2: EDX spectra of HAg_2_S@mSiO_2_@MPDA (a) and HAg_2_S@HMPDA/PEI (b) nanoparticles; Figure S3: TEM images of HAg_2_S@mSiO_2_@MPDA nanoparticles under various PDA reaction times: 0, 20, 40, 60, 90, and 120 min (a–f); Figure S4. TEM images of HAg_2_S@mSiO_2_@MPDA nanoparticles with the addition of various amounts of NH_3_·H_2_O: 25, 50, 100, 150, 200, and 300 μL (a–f); Figure S5: UV-Vis spectra (a) and fluorescence spectra (b) of different samples; Table S1: Fitting results for release profiles of DOX from HAg_2_S@HMPDA/LA-DOX in different release environments.
