# Supplementary material for: Construction of Double-Shelled Hollow Ag2S@Polydopamine Nanocomposites for Fluorescence-Guided, Dual Stimuli-Responsive Drug Delivery and Photothermal Therapy
Source: Nanomaterials (Basel). 2022 Jun 15;12(12):2068. doi: 10.3390/nano12122068 (PMC9230703; doi:10.3390/nano12122068)
Supplement: Supplementary file 1 [file nanomaterials-12-02068-s001.zip › nanomaterials-1760358-supplementary.pdf]

nanomaterials

Supplementary Materials

# Construction of Double-Shelled Hollow $Ag_2S$@Polydopamine Nanocomposites for Fluorescence-Guided, Dual Stimuli-Responsive Drug Delivery and Photothermal Therapy

**Minjie Gao [1], Zehua Han [1], Xu Zhang [2,*], Xueyan Zou [1], Lichao Peng [1], Yanbao Zhao [1] and Lei Sun [1,*]**

1 Engineering Research Center for Nanomaterials, Henan University, Kaifeng 475004, China; gaominjie8@126.com (M.G.); hanzehua0825@163.com (Z.H.); zouxueyan@henu.edu.cn (X.Z.); plc@henu.edu.cn (L.P.); zhaoyb902@henu.edu.cn (Y.Z.)
2 School of Pharmacy, Henan University, Kaifeng 475004, China
* Correspondence: zhangxu@henu.edu.cn (X.Z.); sunlei@henu.edu.cn (L.S.)

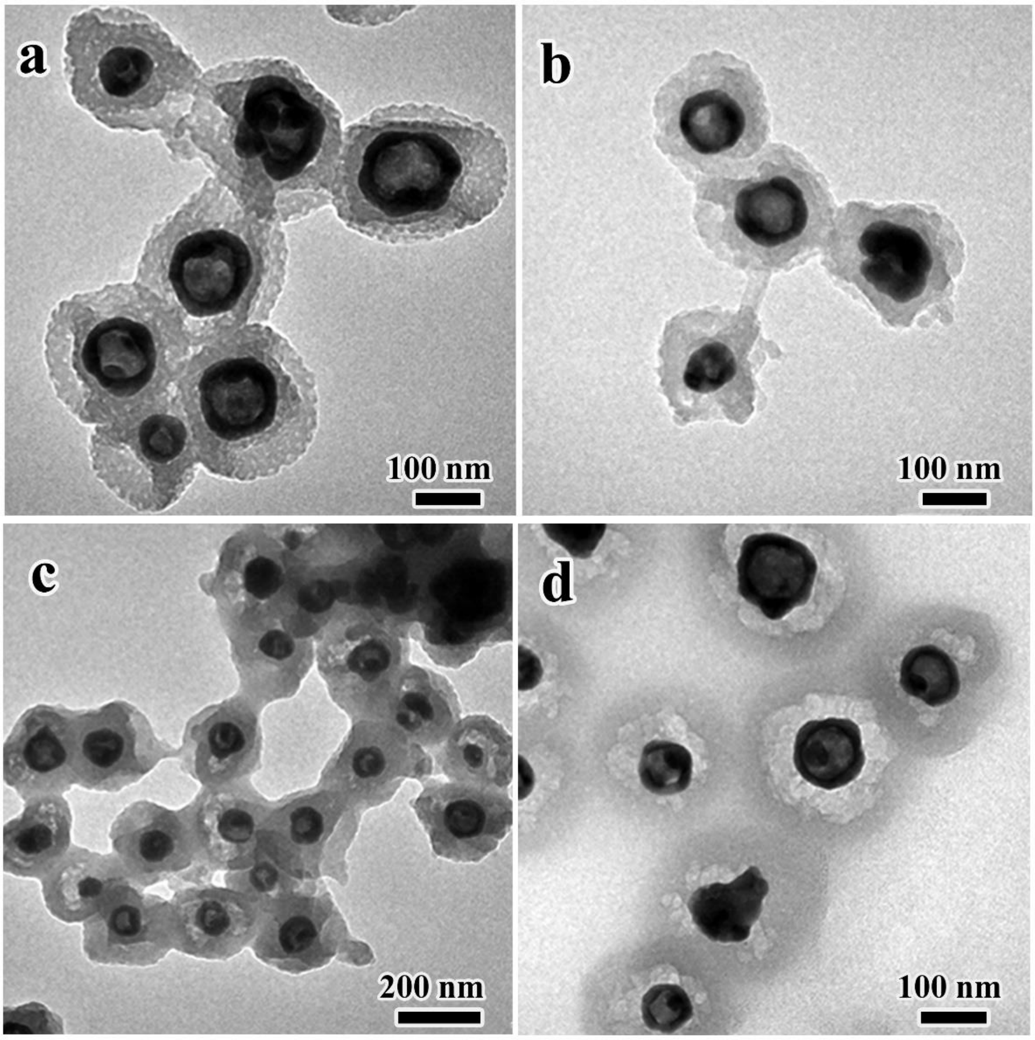


**Figure S1.** TEM images of $HAg_2S$@HMPDA obtained after the etching with different concentrations of $Na_2CO_3$: (**a**–**d**) 0.05, 0.10, 0.20, 0.40 mmol/L.

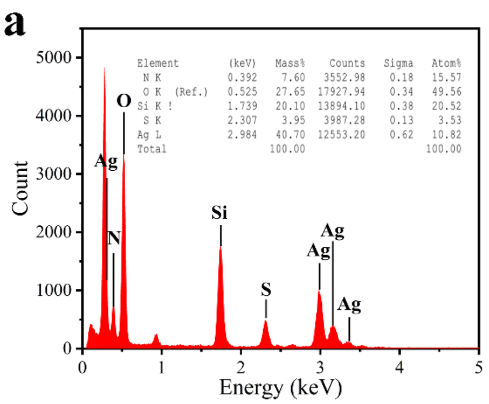


**Figure S2.** EDX spectra of $HAg_2S@mSiO_2@MPDA$ (**a**) and $HAg_2S@HMPDA/PEI$ (**b**) nanoparticles.

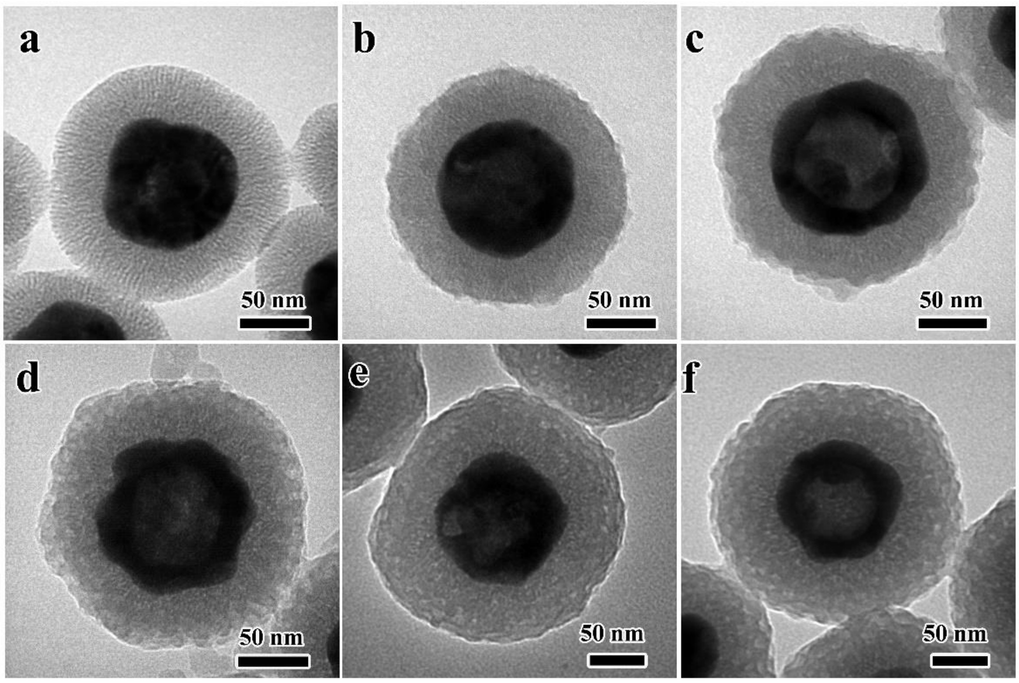


**Figure S3.** TEM images of $HAg_2S@mSiO_2@MPDA$ nanoparticles under various PDA reaction times: 0, 20, 40, 60, 90 and 120 min (**a**–**f**).

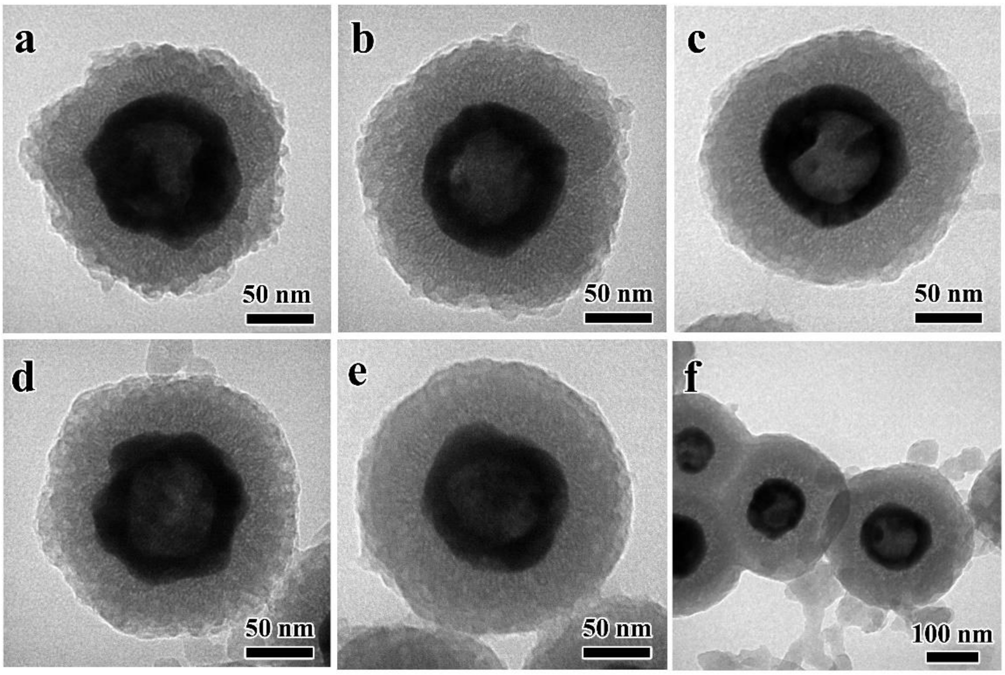


**Figure S4.** TEM images of $HAg_2S@mSiO_2@MPDA$ nanoparticles with various addition of $NH_3 \cdot H_2O$: 25, 50, 100, 150, 200 and 300 μL (**a**–**f**).

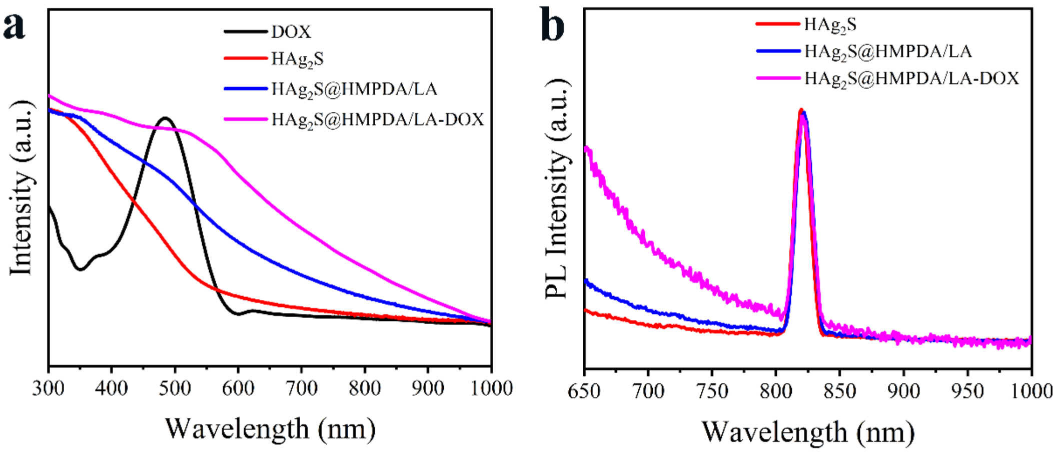


**Figure S5.** UV-Vis spectra (**a**) and fluorescence spectra (**b**) of different samples.

**Table S1.** Fitting results for release profiles of DOX from $HAg_2S@HMPDA/LA-DOX$ in different release environments.

| Release Environment | | Zero-Order Model | | First-Order model | | Higuchi Model | |
|---|---|---|---|---|---|---|---|
| | | Equation | $R^2$ | Equation | $R^2$ | Equation | $R^2$ |
| pH | 7.4 | $Q_t = 0.29t + 13.49$ | 0.2708 | $Q_t = 22.61(1 - e^{-0.28t})$ | 0.9891 | $Q_t = 3.06t^{1/2} + 7.96$ | 0.5701 |
| | 6.5 | $Q_t = 0.39t + 19.86$ | 0.2963 | $Q_t = 31.33(1 - e^{-0.33t})$ | 0.9877 | $Q_t = 4.07t^{1/2} + 19.76$ | 0.5412 |
| | 5.5 | $Q_t = 0.60t + 30.88$ | 0.2395 | $Q_t = 48.80(1 - e^{-0.32t})$ | 0.9904 | $Q_t = 6.31t^{1/2} + 12.43$ | 0.5372 |
| Temperature / °C | 25 | $Q_t = 0.22t + 8.02$ | 0.3145 | $Q_t = 15.00(1 - e^{-0.22t})$ | 0.9874 | $Q_t = 2.15t^{1/2} + 4.19$ | 0.6080 |
| | 37 | $Q_t = 0.29t + 13.49$ | 0.2708 | $Q_t = 22.61(1 - e^{-0.28t})$ | 0.9891 | $Q_t = 3.06t^{1/2} + 7.96$ | 0.5701 |
| | 42 | $Q_t = 0.56t + 30.80$ | 0.2194 | $Q_t = 47.29(1 - e^{-0.35t})$ | 0.9875 | $Q_t = 5.97t^{1/2} + 19.76$ | 0.5133 |
